# A variant of a type V lateral clavicle fracture involving a posteriorly displaced medial segment. A case report

**DOI:** 10.1186/1758-2555-4-47

**Published:** 2012-12-12

**Authors:** Thomas P Goss, Xinning Li

**Affiliations:** 1 University of Massachusetts Medical Center, Worcester, MA, USA

## Abstract

The clavicle connects the shoulder girdle to the axial skeleton, providing support and mobility for optimal upper extremity function. Fractures of the clavicle account for up to 4% of all fractures and comprise up to 44% of all injuries to the shoulder girdle. We present a 63-year-old female patient who suffered what appeared to be a minimally displaced Type V lateral clavicle fracture after a fall as evidenced by an anteroposterior shoulder radiograph. However, an axillary projection demonstrated the proximal segment to be posteriorly displaced and buttonholed through the trapezius musculature with tenting of the skin. The patient underwent an open reduction and Kirschner wire fixation of the fracture with complete healing, subsequent removal of the hardware and return to her previous level of function six months following surgery. After an extensive literature search, we believe this is the first case report documenting a variant of a Type V lateral clavicle fracture, specifically with significant posterior displacement of the proximal segment, mimicking a Type IV AC separation. This fracture pattern is unstable and represents a double disruption of the superior shoulder suspensory complex. Surgical management was successful in returning our patient back to her previous activity of daily living.

## Introduction

The clavicle connects the shoulder girdle to the axial skeleton, providing support and mobility for optimal upper extremity function. Fractures of the clavicle account for up to 4% of all fractures and comprise up to 44% of all injuries to the shoulder girdle [1-3]. These fractures can be separated into three varieties according to anatomic location (Allman’s classification) [4]. The majority occur in the middle third of the clavicle with about 17% involving the lateral third and less than 3% involving the medial third [3]. Fractures of the lateral third of the clavicle can be subdivided into five types. The first three types were originally described by Dr. Neer and types IV and V were added later. Type I injuries are nondisplaced with the coracoclavicular (CC) ligament attached to the medial segment. Type II injuries are separated into IIA and IIB. In the type IIA injury, the CC ligament is not torn and attached to the lateral segment while the medial segment is displaced superiorly. Type IIB fracture consists of a torn conoid ligament while the trapezoid ligament is attached to the lateral fragment, thus the medial segment is displaced. Type III injuries involve the articular surface of the lateral clavicle. Type IV injuries are pediatric periosteal sleeve fractures. Type V are comminuted lateral clavicle fractures with the CC ligament intact but attached to one of the fracture fragments which is detached from the proximal or medial segment [5-7].

We present a 63-year-old female patient who appeared to suffer a minimally displaced Type V lateral clavicle fracture. However, an axillary projection demonstrated the proximal segment to be posteriorly displaced, possibly protruded through the trapezius muscle. In this study, we describe the first report of a case documenting a variation of a type V lateral clavicle fracture, specifically with significant posterior displacement of the proximal segment, mimicking a type IV acromioclavicular (AC) joint separation. This fracture pattern also represents a double disruption of the superior shoulder suspensory complex [8]. The clinical presentation, radiographs, and surgical management are presented. The patient was informed that the data, clinical, and intraoperative pictures concerning her case would be submitted for publication and she has consented. This case report was exempt from the IRB.

## Case report

**Figure 1:**
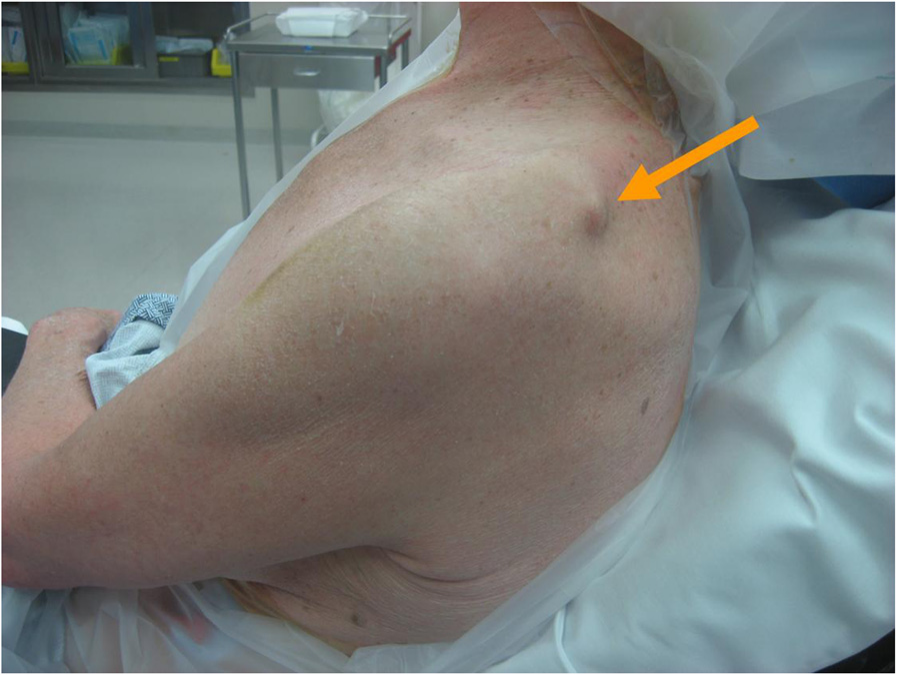
A bony prominence noted on physical inspection of the patient’s shoulder in the region of the supraspinatus muscle with apparent protrusion through the trapezius muscle.

**Figure 2:**
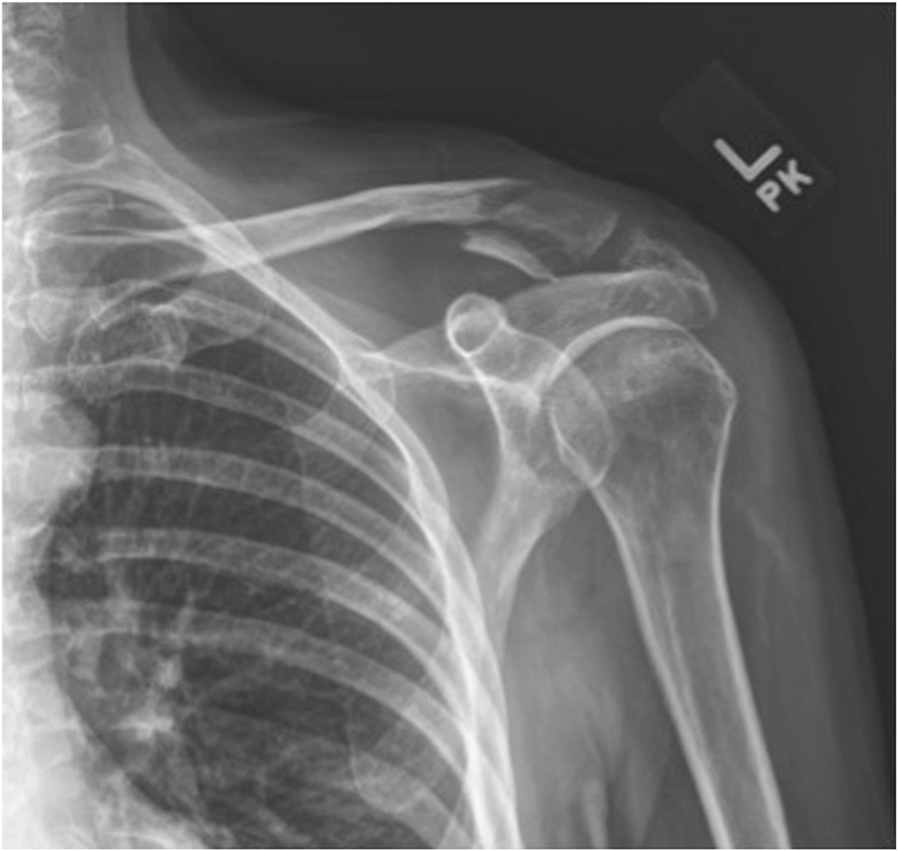
**Anteroposterior radiograph of the left shoulder demonstrating a Type V lateral clavicle fracture with slight superior displacement of the proximal segment.** The CC ligament was intact and attached to the inferior clavicle fracture fragment.

**Figure 3:**
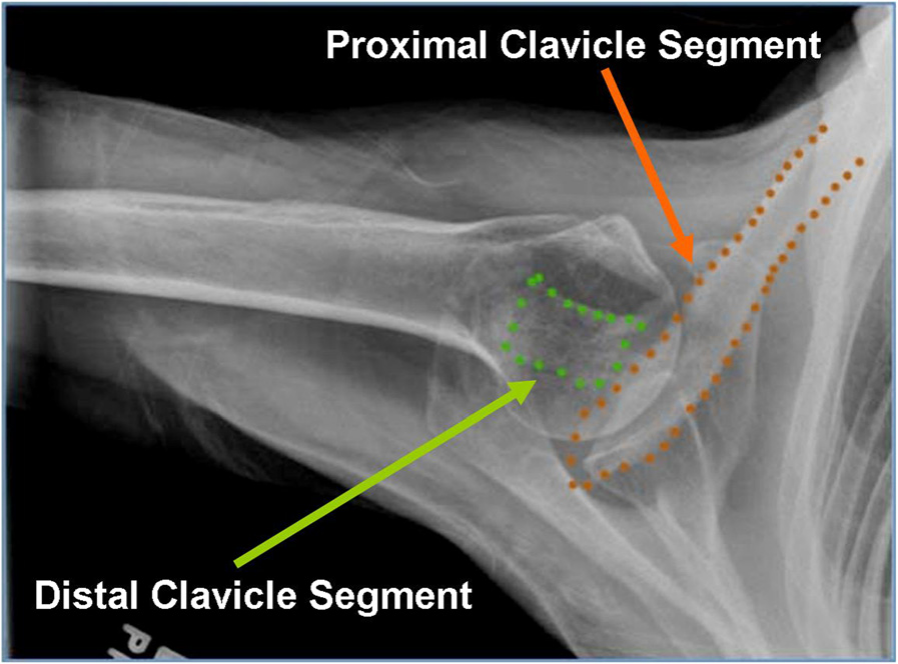
An axillary radiograph of the left shoulder showing the distal clavicular segment aligned with the acromion but the proximal segment significantly displaced posteriorly.

A 63-year-old right hand dominant female sustained a fall injuring her left shoulder. She was initially seen in a local emergency room (ER) on the day of the injury. An anteroposterior radiograph was performed in the ER and the diagnosis of a lateral clavicle fracture was made with sling and swathe as the treatment. Orthopedically, the patient was seen in follow-up two weeks thereafter in clinic. On physical inspection, a bony prominence was noted in the region of the supraspinatus muscle, seemly protruding through the trapezius muscle (Figure 1). The patient was neurovascularly intact with full motor function and normal sensation to light touch over the left upper extremity. Both anteroposterior and the axillary radiographs were obtained. The anteroposterior radiograph (Figure 2) of her shoulder revealed a Type V fracture of the lateral clavicle in what appeared to be in acceptable position, however, the axillary projection showed the distal end of the proximal clavicular segment to be significantly posteriorly displaced (Figure 3).

**Figure 4:**
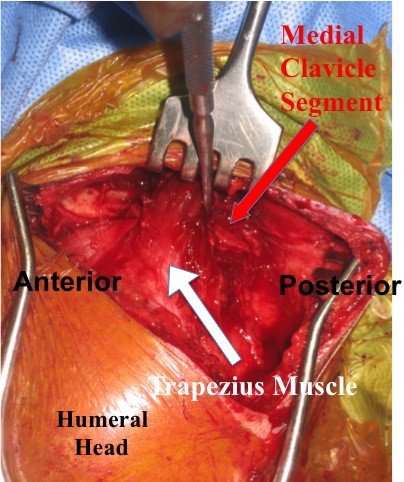
Intraoperative digital photograph demonstrating the posteriorly displaced proximal clavicular segment buttonholed through the trapezius muscle.

**Figure 5:**
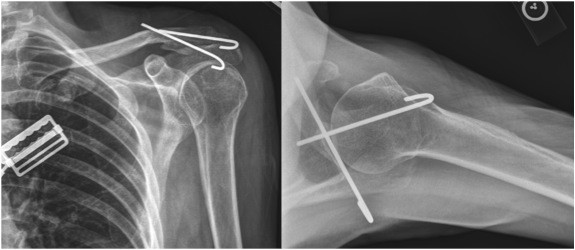
**Postoperative AP and lateral radiographs of the left shoulder.** The proximal clavicular segment is reduced relative to the distal segment and stabilized with two K wires. Sutures were also used in a figure of eight fashion to create tension band constructs. The K wires were bent to prevent migration.

**Figure 6:**
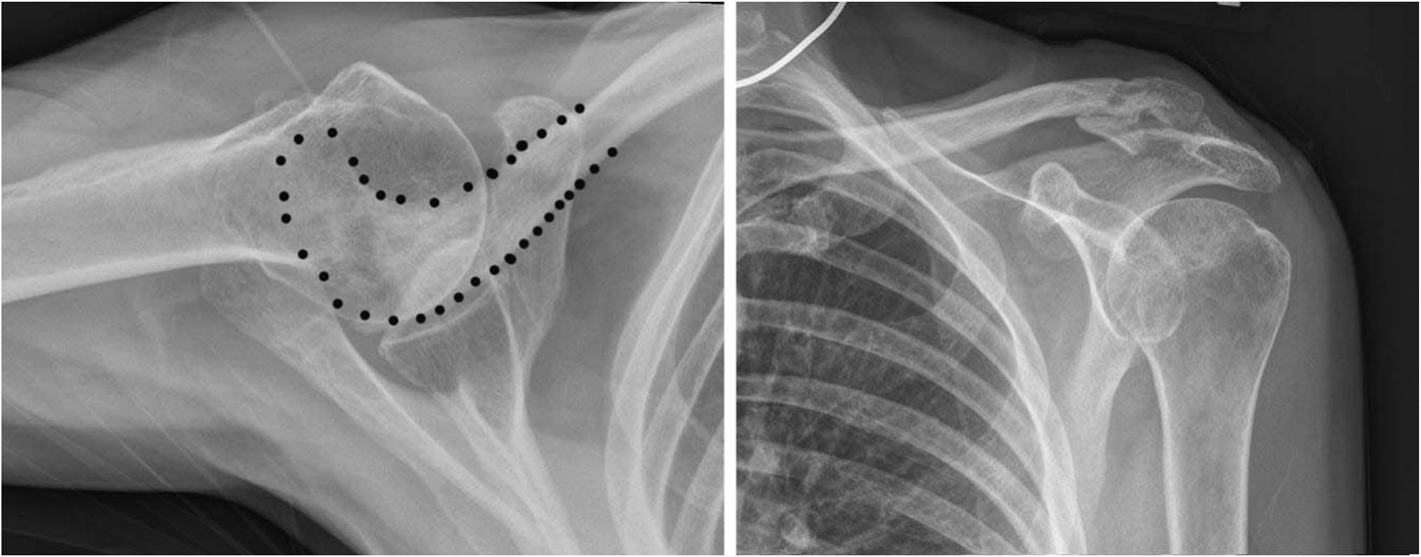
AP and axillary radiographs taken 6 months postoperatively after hardware removal showing good bone healing with near anatomic alignment at the previous fracture site.

The patient was subsequently taken to the operating room at which time the lateral clavicle, AC joint, and acromial areas were surgically exposed. The proximal clavicular segment was indeed posteriorly displaced and buttonholed through the trapezius muscle (Figure 4). The segment was extricated from the muscle and reduced relative to the distal clavicular segment. The inferior bone fragment of the fracture was still attached to both the conoid and trapezoid (CC) ligaments. Internal fixation was provided by two smooth K-wires. One was passed through the proximal aspect of the acromial process and into the intramedullary canal of the proximal segment exiting through its superior cortex. The 2^nd^ K-wire was passed through the distal aspect of the acromial process across the AC joint, and down the intramedullary canal of the distal and proximal clavicular segments exiting out the posterior cortex of the proximal segment. Number 5 Ethibond sutures were then passed around each K-wire in a figure-of-eight fashion to create tension band constructs and secure fixation. The lateral ends of the K-wires were bent to prevent migration and left beneath the skin. The wound was closed with xeroform dressing wrapped around each K-wire. Figure 5 shows the postoperative AP and axillary radiographs. At 6 weeks the K-wires were removed, functional use of the shoulder was encouraged, and physical therapy was begun and designed to regain maximal shoulder range of motion and strength. At the final follow up appointment (1 year post-op), the patient was doing well and had returned to full activity. On physical examination, she had 165 degrees of forward flexion, 45 degrees of external rotation, 110 degrees of abduction and internal rotation to the high part of her back, as well as active elevation of her arm to well above the horizontal. These measurements were very similar to the contralateral upper extremity. A six-month postoperative radiograph is seen in Figure 6.

## Discussion

Fractures of the clavicle are common injuries [1-3]. Most involve males, about 80% involve the middle third and adults are more likely to have a displaced fracture than children [2]. There are two peaks in the incidence of clavicle fractures: the first is in males less than thirty years of age and the second is in females over the age of eighty years [3,9,10]. Most fractures of the middle third are acceptably displaced, whereas fractures involving the medial and lateral thirds tend to be non-displaced [3]. As noted earlier, five types of lateral clavicle fractures have been described in the orthopaedic literature [5-7]. In the Type V variety; the lateral clavicle fracture is comminuted, however the CC ligament (conoid and trapezoid) is intact but attached to an intermediate inferior clavicular fragment, resulting in its functional detachment from the proximal segment [7].

The superior shoulder suspensory complex (SSSC) is comprised of the glenoid, coracoid, and acromial processes, the CC ligament, the lateral clavicle, and the AC joint. The SSSC is therefore a bone and soft tissue structure, which provides considerable stability and some mobility to the shoulder girdle. A disruption of two of the components of the SSSC results in a potentially unstable anatomic situation that can have adverse healing and long term functional consequences [8,11]. Both Type II and V lateral clavicle fractures represent a double disruption of the superior shoulder suspensory complex (two disruptions of the clavicular-scapular linkage), characterized by a lateral clavicle fracture and a detachment of the coracoclavicular ligament from the proximal segment. Consequently, a strong case can be made for simplifying the classification scheme by eliminating the Type V variety and considering such injuries as a Type II variant. As a double disruption of the SSSC, a potentially unstable anatomic situation is present -- the scapular/distal clavicular segment can displace inferiorly and to a lesser degree the proximal clavicular segment can displace superiorly. If displacement is significant, there is the potential for a nonunion occurring at the clavicular fracture site and a surgical ORIF must be considered. In our patient, however, displacement of the proximal segment was posterior with buttonholing through the trapezius muscle (a situation reminiscent of a Type IV AC joint separation). The mechanism of injury most likely included either an anterior to posterior directed force applied to the proximal segment or a posterior to anterior directed force applied to the distal segment or a combination of both [6,11-14]. Many different surgical techniques have been described in the literature for the treatment of significantly displaced lateral clavicle fractures [5,9,15-18].

There are two case reports in the literature describing a posteriorly displaced proximal clavicular segment entrapped within the trapezius muscle associated with a lateral clavicle fracture. Both involved pediatric patients. Itokazu et al. [19] reported an 11-year-old child with this particular injury pattern after a fall from a bicycle who underwent open reduction with Kirschner wire fixation and returned to previous activity at the final follow up. A closed reduction under general anesthesia and non-operative management was also successful in a 13-year-old hockey player with this particular injury pattern. The authors concluded that patients with an open physis and a thick periosteum, a closed reduction could be successful in the treatment of a lateral clavicle fracture with posterior displacement of the medial segment [20]. In our adult patient, however given the significant posterior displacement through the trapezius muscle with tenting of the skin by the proximal segment, a surgical open reduction and internal fixation was necessary to prevent a non-union and persistent discomfort/dysfunction from occurring. Kirschner wire fixation of lateral clavicle fractures has been associated with pin breakage and proximal migration [21]. In our patient, the wires were bent, left beneath the skin and removed when callus was seen on follow-up radiographs. It is essential to bend the K-wire at the end to prevent migration. Another key component of our case is obtaining the axillary radiograph view to make this diagnosis. It is very important to get both the AP and axillary view on lateral clavicle fractures to make sure that the medial segment is not displaced posteriorly before considering treatment options.

To the best of our knowledge, this is the first report of an adult patient with a variant of the type V lateral clavicle fracture and the proximal clavicular segment displaced posteriorly and entrapped within the trapezius muscle. The patient did well and returned to her previous functional status six months following open reduction and Kirschner wire fixation. At the one-year follow-up, the patient continued to do well without any complications.

## Conclusions

In patients sustaining a fracture of the lateral third of the clavicle, both anteroposterior and axillary radiographic views must be obtained given that the AP projection may look to be quite acceptable, while the axillary view may reveal the true nature of the injury. Our patient presented with a type V lateral clavicle injury with the proximal clavicular segment posteriorly displaced and buttonholed through the trapezius muscle. We believe type V lateral clavicle fractures should be considered a type II variant as both represent a double disruption of the superior shoulder suspensory complex (SSSC). Such double disruptions represent a potentially unstable anatomic situation, requiring a surgical open reduction and internal fixation if significant displacement is present as was the case with our patient.

## Competing interests

Both authors declare no conflicts of interest pertaining to this manuscript.

## Authors’ contributions

Both TG and XL are responsible for the concept, data collection, follow-up of the patient, writing/drafting of the manuscript, critical revisions and also the figures associated with this case report. Both authors read and approved the final manuscript.
